# Accounting for Time‐Varying Confounding in a Self‐Controlled Case Series of Fluoroquinolone Treatment for Uncomplicated Urinary Tract Infections and Risk of Collagen‐Related Events

**DOI:** 10.1002/prp2.70124

**Published:** 2025-05-21

**Authors:** Anna Schultze, Shinyoung Ju, Myriam Drysdale, George Mu, Fanny S. Mitrani‐Gold, John Logie

**Affiliations:** ^1^ London School of Hygiene and Tropical Medicine London UK; ^2^ GSK Brentford UK; ^3^ GSK Collegeville Pennsylvania USA

## Abstract

We report findings from three SCCS conducted to quantify the association between fluoroquinolone (FQ) use for the treatment of uncomplicated urinary tract infection (uUTI) and tendon rupture, retinal detachment, and uveitis. Female patients aged ≥ 12 years old in the Optum Clinformatics Data Mart database with an outcome of interest and exposure to either oral FQ or trimethoprim/sulfamethoxazole (SXT) in the 5 days following a newly reported uUTI during the study period (01/01/2011–02/10/2019) were included. We considered a 90‐day risk window for each outcome following drug exposure. Incidence rate ratios (IRR) and 95% confidence intervals (CI) were estimated using conditional Poisson regression, adjusting for age and calendar time, incorporating SXT as an active comparator. We found little evidence of an association between FQ use compared to SXT and any of the outcomes of interest (tendon rupture: IRR = 1.01, 95% CI = 0.91–1.21; retinal detachment: IRR = 0.99, 95% CI = 0.86–1.14; and uveitis: IRR = 1.09, 95% CI = 0.97–1.22). Incorporating the active comparator using two different methods did not change conclusions. The lack of evidence for an association between short‐term use of FQ and the comparator antibiotic (SXT) for the treatment of uUTI and collagen‐related events also implies that there was no marked association between uUTI and these outcomes. However, power was limited, and a small increased risk cannot be ruled out. Using active comparators in SCCS can improve the robustness of studies of antibiotics and adverse events.

## Graphical abstract

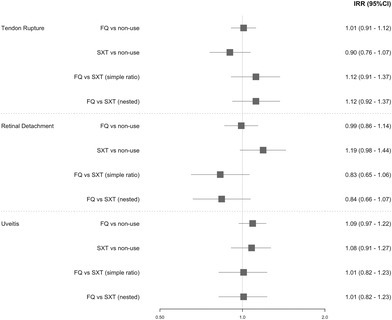

## 1 Introduction

Self‐controlled case series (SCCS) designs are often used to evaluate medication safety as, by design, they control for all measured and unmeasured confounders that do not vary over time [1]. However, time‐varying confounding is still a challenge, particularly when potential confounders are unmeasured or difficult to adjust for using multivariable models. A recent extension of the SCCS uses an active comparator to control for potential time‐varying confounding by indication [2]. Our objective was to apply this design to study the association between fluoroquinolone (FQ) treatment for uncomplicated urinary tract infection (uUTI) and collagen‐related adverse events compared to trimethoprim/sulfamethoxazole (SXT). Unmeasured confounding is a particular challenge when studying this question, as the uUTI that resulted in FQ treatment might increase the risk of the collagen‐related adverse events we were interested in.

## 2 Methods

We report findings from three SCCS conducted to quantify the association between FQ use for uUTI treatment and collagen‐related adverse events of interest, namely, tendon rupture, retinal detachment, and uveitis. As the infection (uUTI) leading to FQ treatment is transient and therefore varies over the observation time, it would not be inherently controlled by the application of a standard SCCS. We therefore used SXT as an active comparator. We selected cases of female patients aged ≥ 12 years old in the Optum Clinformatics Data Mart (CDM) database with an outcome of interest (further details in Supporting Information) and exposure to either oral FQ or SXT in the 5 days following a new reported uUTI (algorithm and ICD‐10 codes described in Supporting Information) during the study period (01/01/2011–02/10/2019). Optum CDM contains data on individuals from the United States insured by commercial and Medicare Advantage plans [3, 4]. Where there were multiple codes relating to the same outcome event, a period of 90 days was used to define unique outcome occurrences. The exposure of interest was, specifically, FQ use after a uUTI. This was done to ensure that the risk periods for both the drug of interest and the comparator were more homogenous in terms of underlying disease severity. uUTI is one of the most common indications for FQ use, so studying this specific indication was of particular interest [5]. To further ensure that the drug of interest and comparator were used for a similar amount of time, we only considered drug exposures with 3–10 recorded days of supply (the recommended treatment duration for uUTI). The days' supply is recorded as a variable at the time of the prescription in the Optum CDM.

**FIGURE 1:**
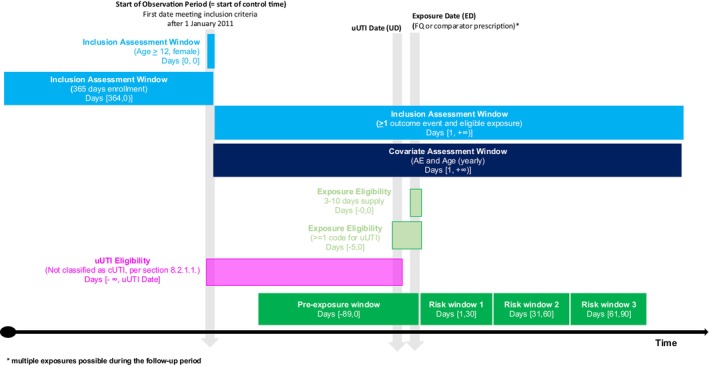
Illustration of key definitions used in the SCCS design.

Among the cases, we then constructed a 90‐day risk window for each outcome following drug exposure, and applied a 90‐day pre‐exposure window to account for potential violations of the core SCCS assumption that the occurrence of the outcome does not alter the future probability of exposure [6]. Overlapping risk periods were treated as a separate level of the exposure variable. All other observation time was used as reference time (Figure 1); details of how the risk periods were specified are provided in the Supporting Information.

## 3 Statistical Analysis

As in a standard SCCS, the incidence rate ratio (IRR), with 95% confidence intervals (CI), was estimated using conditional Poisson regression, adjusting for two time‐updated variables: age and calendar time (both in years). We then incorporated the active comparator in two ways following [2]: firstly, as a simple ratio and secondly, as a nested regression model. The simple ratio method was implemented by considering two separate case series for each outcome: one with all patients exposed to FQ, and one with all patients exposed to the comparator (SXT). Patients who had exposure to both drugs during their follow‐up time could contribute information to both of these case series, but overlaps between FQ and SXT exposure were removed from the baseline time. We then calculated an “active comparator rate ratio” by dividing the IRR from a given period in the FQ case series with that from the corresponding period in the SXT case series. 95% CIs were constructed using the Wald method. The nested regression model is fitted on a single case series containing data from patients exposed to either the drug of interest or the comparator. An additional variable, representing exposure to either drug, is then constructed. The nested model uses this new variable as the main exposure and includes an interaction term between this and the drug of interest to estimate the same active comparator rate ratio as specified above. Data management was conducted using SAS Studio 3.81, and analyses were conducted using Stata 14.

## 4 Sensitivity Analyses

We conducted several sensitivity analyses. Firstly, we divided the 90‐day risk periods into 30‐day intervals to investigate whether the original 90‐day period may have underestimated a more rapid and transient increase in the risk of a particular adverse event. Secondly, we conducted a post hoc analysis only including calendar years with both an outcome and a risk period.

## 5 Results

**FIGURE 2:**
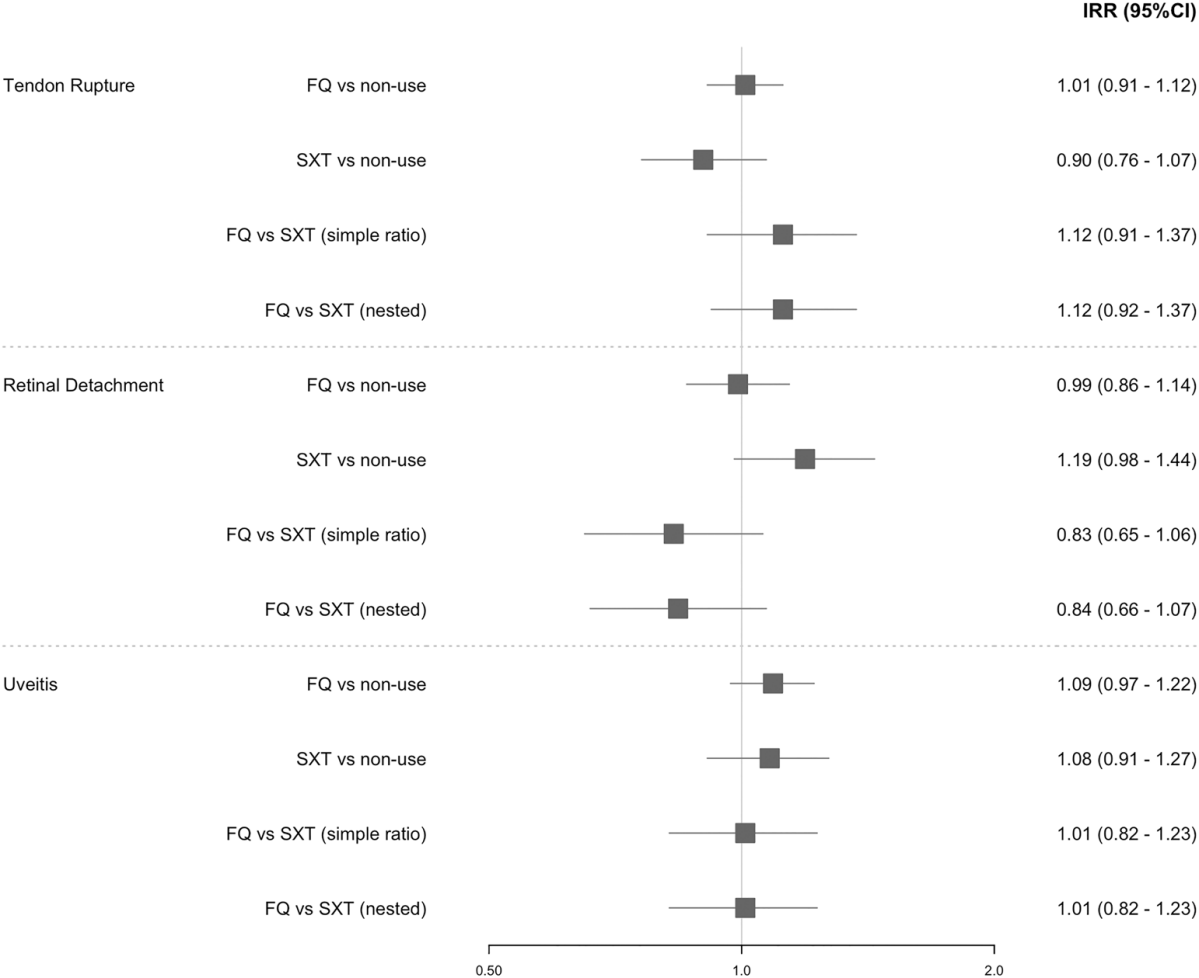
Adjusted incidence rate ratios with 95% confidence intervals (95% CIs) for fluoroquinolones (FQ) versus trimethoprim/sulfamethoxazole (STX) for the Day 1–90 risk period.

There were 9010 women in the tendon rupture case series, 5383 in the uveitis case series, and 2923 in the retinal detachment case series. The median (IQR) ages of the individuals were 61 (21), 64 (26), and 66 (19) at the beginning of the follow‐up in the tendon rupture, uveitis, and retinal detachment case series, respectively. The median (IQR) follow‐ups (years) of the individuals were 6.8 (4.5), 6.2 (4.8), and 6.8 (4.7) in the tendon rupture, uveitis, and retinal detachment case series, respectively. Incidence rate ratios (IRR and 95% CIs) for collagen outcomes can be seen in Figure 2.

There was little evidence of an association between FQ use and any of the collagen‐related AEs of interest, with all 95% CIs including the null. Incorporating the active comparator did not change these conclusions, although the IRR moved somewhat higher for tendon rupture and lower for retinal detachment. There was agreement between the two methods for incorporating the active comparator, and results from the two methodological sensitivity analyses (Table 1) were consistent with the primary analysis.

**TABLE 1:** Active comparator rate ratios with 95% confidence intervals using each method in the sensitivity analyses.

Outcome	Period	FQ		SXT		Simple ratio		Nested	
		IRR	95% CI	IRR	95% CI	IRR	95% CI	IRR	95% CI
Sensitivity #1: finer risk periods									
Tendon rupture	Days 1–30	1.03	0.87–1.21	0.88	0.67–1.16	1.17	0.85–1.61	1.17	0.85–1.61
	Days 31–60	1.02	0.86–1.22	0.77	0.56–1.06	1.33	0.93–1.90	1.33	0.93–1.90
	Days 60–91	0.96	0.79–1.17	1.07	0.81–1.42	0.90	0.64–1.26	0.90	0.64–1.26
Retinal detachment	Days 1–30	0.95	0.75–1.20	1.12	0.81–1.54	0.85	0.57–1.27	0.86	0.57–1.27
	Days 31–60	0.80	0.61–1.04	1.18	0.84–1.64	0.68	0.44–1.04	0.68	0.44–1.04
	Days 60–91	1.25	1.00–1.56	1.28	0.92–1.79	0.97	0.65–1.45	0.98	0.66–1.46
Uveitis	Days 1–30	1.16	0.97–1.39	0.99	0.75–1.30	1.18	0.85–1.63	1.18	0.85–1.64
	Days 31–60	1.06	0.87–1.29	1.01	0.76–1.36	1.04	0.73–1.48	1.04	0.74–1.48
	Days 60–91	1.02	0.83–1.26	1.26	0.96–1.66	0.81	0.57–1.14	0.81	0.57–1.14
Sensitivity #2: restricted observation time									
Tendon rupture	Days 1–90	1.00	0.89–1.13	0.86	0.71–1.04	1.16	0.93–1.46	1.17	0.94–1.47
Retinal detachment	Days 1–90	0.94	0.80–1.11	1.11	0.89–1.39	0.84	0.64–1.12	0.85	0.65–1.12
Uveitis	Days 1–90	1.06	0.93–1.21	1.01	0.84–1.22	1.05	0.83–1.32	1.05	0.83–1.32

## 6 Discussion

In this study, we used several strategies to control for potential time‐varying confounding in an SCCS of the association between FQ use for uUTI and collagen‐related events of interest. Overall, there was limited evidence for an association between the use of either FQ or SXT with any of the outcomes, and as a result, the incorporation of the comparator (SXT) made relatively little difference. The simple ratio and nested regression method for incorporating the active comparator gave similar results.

The association between FQ and tendon injury is well‐established [7, 8], but observational data for other collagen‐related outcomes are less consistent. A systematic review investigating the association between FQ and retinal detachment found an increased risk in cohort studies, but not in SCCS, which are expected to have better control for time‐invariant confounders [9]. This implies that a key reason for previously discrepant findings may be unmeasured confounding which, in this study, we attempted to address in three ways: Firstly, by restricting to exposures following a uUTI to remove heterogeneity in the study population, which could potentially contribute to confounding by indication [10]; secondly, by using an SCCS design to control time‐invariant confounding [11]; and finally, by incorporating an active comparator [2]. Despite our hypothesis that we may observe an increased risk of our outcomes for both the drug of interest and comparator due to time‐varying confounding by indication, we found null results in all case series, and as a result, the application of the active comparator made little difference to our conclusions. It might be that the indication studied here—uUTI—has a weaker association with collagen‐related AEs than other infections.

The lack of association between FQ and tendon rupture observed here could be due to the restriction of exposures to those following a uUTI, meaning that the study population was generally relatively young (with median age ranging from 61 to 66 depending on the case series), the average days of supply of the exposures was short (3–10 days) and the FQ drugs included were specifically those indicated for use in uUTI rather than all FQs. We also studied all nontraumatic tendon ruptures (Achilles + other tendons) as this was one of our prespecified outcomes of interest, and the strongest signals in the published literature have been for Achilles tendon rupture (nontraumatic). Studying all tendon ruptures also allowed us to ascertain events using CPT procedure codes for the repair of a tendon rupture, in addition to ICD codes, to capture more outcomes, as these are not location specific. Risks have also been reported to increase with cumulative exposure duration and to be particularly high in the presence of concomitant corticosteroid exposure [12]: in this study, we excluded patients receiving immunosuppressive therapies. A recent Taiwanese cohort study of patients treated specifically for UTI reported no association between FQ use and the absolute risk of aortic aneurysm/detachment and suggested this may be due to the restriction of their study to a population with underlying infections of similar severity [13]. However, we want to stress that with 95% CIs indicating our data is consistent with effect sizes between 0.91 and 1.37, we cannot rule out a small increase in the risk of tendon rupture in this population.

Methods for incorporating active comparators in SCCS have only been recently developed, and experience in the application of the methods is therefore relatively limited. Chui et al. used the method to evaluate the association between proton pump inhibitors and myocardial infarction [14]. They found a strong association between both the drug of interest and the outcome, and the comparator (H2 agonists) and the outcome. The authors interpret this as potential time‐varying protopathic bias [15]. However, their manuscript only applied the simple ratio method and could not comment on the agreement between the simple ratio and nested regression approaches. Our finding that the different methodologies for incorporating the comparator gave highly consistent results is reassuring, as the original evaluation found some differences when applied in an SCCS design [2].

Our study has several strengths. As mentioned above, we attempted to control for confounding in numerous ways and conducted several sensitivity analyses. The fact that the population was homogenous with the same indication required for both FQ and SXT risk periods will have minimized differences in the underlying disease severity. We also had access to a large dataset, although power still appeared relatively low with wide CIs for many of our outcomes. The study also had some notable limitations. Firstly, there may be some misclassification of both the exposure and outcomes. The fact that we required uUTI diagnoses to be followed by a relevant prescription should give us some confidence in the sensitivity of our algorithm, but we may still have excluded some relevant prescriptions if UTI diagnoses were not recorded. Due to small patient numbers, we were also unable to study specific FQs or stratify on the basis of dose received. This is important to acknowledge, as it means we were not able to evaluate any potential dose–response effect, which has previously been reported for FQs and tendon rupture [7]. Standard limitations for observational analyses of US claims databases apply; specifically, we were not able to account for any potential exposure to antibiotics during hospitalizations, as these were not recorded, and our findings may not be generalizable to the target population of all women experiencing uUTI.

## 7 Conclusion

In this study, we found no evidence of an association between short‐term use of FQ and a comparator antibiotic (SXT) for the treatment of uUTI and collagen‐related events. The lack of association between FQ compared to the unexposed reference time also implies that there was no confounding by indication, or in other words, there was no marked association between uUTI and these outcomes. However, power was limited, and the results from this study also do not exclude a potential association between longer use of FQ and collagen‐related events. Using active comparators in SCCS holds promise for such studies, and we encourage other researchers to consider a multipronged approach to tackling confounding by indication in their investigations of FQ safety.

## Author Contributions

All authors had access to the study data, take responsibility for the accuracy of the analysis, contributed to data interpretation, reviewed and contributed to the content of the manuscript, and had authority in the decision to submit the manuscript.

## Ethics Statement

This study complied with all applicable laws regarding subject privacy; no direct subject contact or primary collection of individual human subject data occurred. Study results are presented as de‐identified composite analyses. Therefore, informed consent, ethics committee, or Institutional Review Board approval was not required.

## Conflicts of Interest

**A.S**. is an assistant professor at the London School of Hygiene and Tropical Medicine employed on a grant from GSK. **S.J**. is an employee of and shareholder in GSK. **M.D**. is an employee of and shareholder in GSK. **G.M**. is an employee of and a shareholder of GSK. **F.S.M.G**. is an employee of and shareholder in GSK. **J.L**. was an employee of and shareholder in GSK at the time the work was conducted.

## Supporting information


Data S1.

## Data Availability

The data that support the findings of this study were made available to the authors through third‐party license from Optum Clinformatics Data Mart, a commercial data provider in the United States. As such, the authors cannot make these data publicly available due to a data use agreement. Other researchers can access these data by purchasing a license through Optum Clinformatics Data Mart. Inclusion criteria specified in the Methods section would allow other researchers to identify the same cohort of patients used for these analyses. Interested parties may see https://www.optum.com/business/life‐sciences/real‐world‐data/claims‐data.html for more information on Optum Clinformatics Data Mart.
