# Supplementary material for: Accounting for Time‐Varying Confounding in a Self‐Controlled Case Series of Fluoroquinolone Treatment for Uncomplicated Urinary Tract Infections and Risk of Collagen‐Related Events
Source: Pharmacol Res Perspect. 2025 May 21;13(3):e70124. doi: 10.1002/prp2.70124 (PMC12093150; doi:10.1002/prp2.70124)
Supplement: Supplementary file 1 — Data S1. [file PRP2-13-e70124-s001.docx]

**Supplementary Materials**

***Algorithm for defining uUTI***

A UTI was defined as the presence of at least one diagnosis claim (ICD-9-CM/ or ICD-10 diagnosis code) indicative of UTI (acute cystitis with or without haematuria, cystitis unspecified or UTI site not specified).

- - ICD9-CM: 595.0, 595.9, 599.0
  - ICD-10-CM: N30.00, N30.01, N30.90, N30.91, N39.0 35

Of note, the ICD-9/10 codes for UTI are not specific to uncomplicated UTI (uUTI) and complicated UTI (cUTI), therefore we include all relevant codes for UTI and then applied exclusion criteria to exclude cUTI. The following events were considered complicated UTI (cUTI) and used to remove patients with urological abnormalities and complicating UTI comorbidities:

- UTI events recorded in an inpatient setting
- UTI events in patients with ICD-9/10 codes for structural or functional abnormalities of the urinary tract or urological procedures (HCPCS) associated with cUTI on the date of the UTI event or in the prior 364 days
- UTI events in patients who were pregnant at the time of the UTI (defined by pregnancy diagnosis or procedure codes (ICD-9/10 and HCPCS) recorded within the period prior to the UTI event date (as a period of 9 months + 28 days)
  - A 28-day pregnancy washout period was used to confirm that UTI events included in the study occur ≥28-days post end of pregnancy and not residual effects from pregnancy. Any UTI events which occur within 28- days of the end of pregnancy were considered a cUTI.
- UTI events in patients with complicated or uncontrolled diabetes mellitus, as defined by the presence of ≥ one diagnosis code for complicated or uncontrolled diabetes mellitus on the date of the UTI event or in the prior 364 days, and a prescription of a relevant diabetes medication on the date of the UTI event or in the prior 364 days.
- UTIs events in patients treated with intravenous (IV) antibiotic therapy (identified via HCPCS codes) within the 5 days following the initial diagnosis claim. Note- the exposure washout period is for the antibiotics being assessed in this study (of which FQ and TMP-SMX have an IV formulation)
- UTI events in patients with immunosuppression or treatment with immunosuppressive therapy, defined by the presence of ≥ one diagnosis code for immunosuppression on the date of the UTI event or in the prior 364 days, or by a prescription for immunosuppressive therapy on the date of the UTI event or in the prior 364 days.
- Treatment with immunosuppressive therapies
- Treatment with chemotherapy and /or radiation

UTI events appearing anytime on or after the earliest diagnosis date of HIV

***Definitions used for the outcomes of interest***

- **Tendon rupture (≥ 1 of the following ICD9/10 OR CPT codes)**
  - Defined by the presence of an ICD9/10 code for a spontaneous / non-traumatic rupture of ≥ one of the following tendons (Achilles, quadricep, patellar or tibial) OR
  - Defined by the presence of a CPT code for tendon repair (Achilles, quadricep, patellar or tibial) OR
    - ICD9-CM codes: 727.6 (727.60, 727.62-69) (exclude 727.61 which is rotator cuff rupture but doesn’t include non-traumatic classification) OR
    - ICD-10 codes: M66.2- M66.9 (as per CID 892 for spontaneous non-traumatic tendon ruptures) OR
    - Tendon Repair CPT codes: 27380, 27381, 27650, 27652, 27654, 27658, 27659, 27664, 27665 (day +1 to day +90 and absence of these codes in the outcome washout period (90 days prior to the exposure)
- **Retinal detachments**
  - Defined by the presence of an ICD9/10 code for retinal detachment.
    - ICD-9-CM codes: 361.0, 361.00, 361.01, 361.02, 361.03, 361.04, 361.05, 361.2, 361.89, 361.9 OR
    - ICD-10 codes: H330-H332 (including child codes)
- **Uveitis**
  - Defined by the presence of an ICD9/10 code for uveitis.
    - ICD-9-CM codes: 360.12, 363.0,363.00-08, 363.1-15, 363.2, 363.20, 364.0-04, 364.3 OR
    - ICD-10-CM codes: uveitis codes in CID 894

**Detailed Description of and Rationale for using Optum CDM**

The Optum Clinformatics® Data Mart (CDM) database is a de-identified administrative adjudicated health claims database from commercial and medicare (Medicare Advantage) health plans, which incorporates members from all 50 US states. The insurance claims data include all medical (outpatient, emergency department, inpatient) and pharmacy claims submitted for reimbursement on behalf of health plan members. This database accesses commercial and Medicare Advantage claims, and enrolment links patient and physician data to pharmacy and medical claims. Medical claims or encounter data, collected from all available health care sites, are deidentified or anonymized, such that the database is compliant with the Health Insurance Portability and Accountability Act. This database was selected in part because of the ability to assess mortality outside of the hospital setting (a censoring variable in this study). The Optum database uses 6 different data sources, including Centers for Medicare and Medicaid Services (CMS), SSA Death Master File, Facility Claims, Member Coverage data, Optum Electronic Health Records, and external Obituary data, which contribute to the mortality data. This feature made the Optum database more appropriate for the study's methods, which relied on being able to censor patients on death, compared to other claims databases.

**Time Levels and Definitions for SCCS Design**

| ***Time levels*** | ***Definition for FQ*** | ***Definition for SXT*** |
| --- | --- | --- |
| 0 - Baseline | None of the below | None of the below |
| 1 – Pre-exposure Period  (Days -89 to 0) | Pre-exposure for “eligible” FQ prescription (day -89 to day 0) and no overlap with any other eligible or ineligible prescription risk period | Pre-exposure for “eligible” SXT prescription (day -89 to day 0) and no overlap with any other eligible or ineligible prescription risk period |
| 2 – Risk period  (Days + 1 to + 30) | Risk period days +1 to + 30 for “eligible” FQ prescription and no overlap with any other eligible or ineligible prescription risk period | Risk period days +1 to + 30 for “eligible” SXT prescription and no overlap with any other eligible or ineligible prescription risk period |
| 3 – Risk period  (Days + 31 to + 60) | Risk period days +31 to +60 for “eligible” FQ prescription and no overlap with any other eligible or ineligible prescription risk period | Risk period days +31 to +60 for “eligible” SXT prescription and no overlap with any other eligible or ineligible prescription risk period |
| 4 – Risk period  (Days + 61 to + 90) | Risk period days +61 to + 90 for “eligible” FQ prescription and no overlap with any other eligible or ineligible prescription risk period | Risk period days +61 to +90 for “eligible” SXT prescription and no overlap with any other eligible or ineligible prescription risk period |
| 5 – Overlap of Risk Periods | Overlap between the classes (1,2,3, 4) either within or between eligible FQ or SXT | Overlap between the classes (1,2,3, 4) either within or between eligible FQ or SXT |
| 6 – Ineligible Prescriptions  (Days +1 to +90) | Ineligible FQ prescriptions (day +1 to day +90). This includes days overlapping with eligible FQ or SXT. | Ineligible SXT prescriptions (day +1 to day +90). This includes days overlapping with eligible FQ or SXT. |
